# Third Body Wear of UHMWPE-on-PEEK-OPTIMA™

**DOI:** 10.3390/ma13061264

**Published:** 2020-03-11

**Authors:** Raelene M. Cowie, Naveen Manikya Pallem, Adam Briscoe, Louise M. Jennings

**Affiliations:** 1 Institute of Medical and Biological Engineering, School of Mechanical Engineering, University of Leeds, Leeds LS2 9JT, UK; r.cowie@leeds.ac.uk (R.M.C.); speak2manikya@gmail.com (N.M.P.); 2 Invibio Ltd., Invibio Global Technology Centre, Hillhouse International, Thornton-Cleveleys, Lancashire FY5 4QD, UK; abriscoe@invibio.com

**Keywords:** biomaterials, arthroplasty, orthopaedic tribology, experimental simulation, total knee replacement, PEEK-OPTIMA™, UHMWPE, third body wear

## Abstract

PEEK-OPTIMA™ is being considered as an alternative to cobalt chrome (CoCr) in the femoral component of total knee replacements. To date, investigations of ultra-high molecular weight polyethylene (UHMWPE)-on-PEEK have shown an equivalent wear rate to conventional implant materials under standard conditions. In this study, the third body wear performance of UHMWPE-on-PEEK was directly compared to UHMWPE-on-CoCr in a series of pin-on-plate studies using two approaches for third body damage. Damage simulation with particles of bone cement showed a significant (*p* < 0.001), four-fold increase in the mean surface roughness of PEEK plates compared to CoCr. However, wear simulation against the damaged plates showed no significant difference in the wear of UHMWPE pins against the different materials (*p* = 0.59), and a polishing effect by the pin against the PEEK plates was observed. Scratching PEEK and CoCr counterfaces with a diamond stylus to create scratches representative of severe third body damage (4 µm lip height) resulted in a significantly higher (*p* = 0.01) wear of UHMWPE against CoCr compared to PEEK and again, against PEEK plates, polishing by the UHMWPE pin led to a reduction in scratch lip height. This study shows that in terms of its wear performance under third body wear/damage conditions, UHMWPE-on-PEEK differs from conventional knee replacement materials.

## 1. Introduction

Total knee replacement (TKR) is a highly successful procedure with >90% survival at 10 years [1]; however, up to 20% of patients may be dissatisfied with their procedure [2]. When considering how to improve patient satisfaction, the materials from which the implant is manufactured is one potential variable to be considered. PEEK-OPTIMA™ (poly-ether-ether-ketone) has recently been investigated as an alternative to cobalt chrome (CoCr) for use in the femoral component of TKR [3,4,5]. There are a number of potential advantages of using PEEK in this application. For example, the Young’s modulus of the PEEK femoral component (~3.7 GPa) is more similar to that of bone (0.001–20 GPa) than a CoCr femoral component (~210 GPa), giving the potential for a reduction in stress shielding, which can lead to bone resorption particularly behind the anterior flange of the implant and, hence, increase the risk of implant loosening [6]. In addition, an all-polymer implant would be lighter weight than conventional materials and more similar to the weight of the natural joint.

Several in vitro pin-on-plate and whole joint simulation studies have been carried out investigating the wear performance of the PEEK-on-ultra-high molecular weight polyethylene (UHMWPE) bearing couple and have demonstrated an equivalent rate of wear for this bearing couple compared to CoCr-on-UHMWPE for a well-positioned implant tested in a clean environment under standard conditions [3,7,8]. In addition, an in vivo large animal study has also investigated the potential to use PEEK in an all-polymer TKR [9,10]. Despite this animal study being relatively short-term, no problems were encountered with fracture or fixation of the device; there was, however, an inflammatory response but, due to the lack of a control group (metal-on-polyethylene implant), it is not known whether a similar response would occur if conventional TKR materials were implanted in this animal model. Having obtained promising initial results, prior to the clinical adoption of the device, it is important to consider how the implant will respond under a wider envelope of clinically relevant conditions [11]. In this study, the influence of third body wear on UHMWPE-on-PEEK-OPTIMA™ in a simple geometry pin-on-plate configuration has been considered. 

Third body wear can occur when hard particles such as bone cement particles, bone fragments or other particulate materials become trapped between the articulating surfaces of an implant causing damage to the joint surfaces and accelerating implant wear. Two approaches have been taken for the simulation of third body wear of arthroplasty bearing materials. One approach has been to introduce clinically relevant particles such as polymethyl methacrylate (PMMA) cement into the articulating interface [12]; an alternative approach has been to inflict third body damage directly to the surface(s) either by scratching the surface of the implant directly using a diamond stylus, which gives close control of the position and geometry of the scratches [13], or by abrading the component bearing surface producing a random orientation of scratches [14]. There are advantages and limitations to these approaches. Protocols using particles to replicate third body damage can more closely represent the clinical situation; however, controlling the particles trapped between the articulating surfaces can be difficult especially in the knee where the low conforming nature of the articulating surfaces may lead to particles being ejected from the joint. In bearing couples containing polymeric implant materials, if particles become embedded in the polymer, assessing wear of the polymer gravimetrically can be unreliable. Scratching the surface of the implant directly can give more consistent and reproducible damage. Controlling the lip height of the scratches, which is the variable that most influences polyethylene wear in metal-on-UHMWPE configurations, using a method such as a diamond stylus allows the scratch geometry to be optimised to more closely replicate observations of retrieved implants [15]. However, this approach could be considered a less clinically relevant method for simulating third body damage than using particles.

Whilst carrying out whole joint wear simulation studies is optimal, valuable information can be gained from pin-on-plate studies. By simplifying component geometries and applied loads and motions, the influence of individual variables can be investigated [15]. The aim of this study was to investigate the influence of third body damage on the wear of UHMWPE-on-PEEK-OPTIMA™ in simple geometry pin-on-plate wear simulation. Third body damage was simulated using two approaches: (1) using particles of PMMA cement and (2) scratching the implants directly using a diamond stylus. When considered together, the outcomes of these two approaches can give a better understanding both of how third body particles can damage the articulating surfaces, and how different magnitudes of damage can influence wear. For all the studies, the wear of UHMWPE-on-PEEK was directly compared to conventional knee implant materials, UHMWPE-on-CoCr, which were tested in parallel. It was hypothesised that, because of the different material properties of PEEK compared to CoCr, the third body wear behaviour of the two material combinations would differ.

## 2. Materials and Methods 

The studies were split into two phases, first carrying out damage simulation (using each method) before determining the wear factors of UHMWPE against the damaged surfaces. This two-phase approach has been adopted in previous third body wear simulation studies [15,16].

### 2.1. Materials

The pins used were GUR 1020 UHMWPE (conventional, non-sterile) machined into a truncated cone with either 3 mm or 8 mm flat contact face for damage or wear simulation, respectively. The plates were either injection moulded, implant grade, unfilled (natural) PEEK-OPTIMA™ (Invibio Ltd, UK), initial surface roughness (Ra) ~0.02 µm or CoCr, polished to an initial Ra < 0.01 µm. To create damage with particles, Palacos R + G PMMA cement (Heraeus, Germany) was mixed and cured in a block as per the manufacturers’ instructions before turning and crushing with a mortar and pestle to create particles, which were sieved within a size range of 500–1000 µm diameter. The particle size range used was within a clinically relevant range [17].

### 2.2. Methods

#### 2.2.1. Damage Simulation: Third Body Damage with PMMA Cement Particles

**Figure 1:**
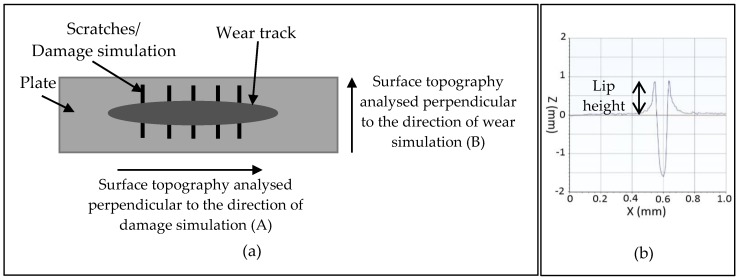
(**a**) Schematic showing the respective directions of the damage simulation and wear simulation; (**b**) schematic showing the profile of a 1 µm scratch created with a diamond stylus and the lip height measurement taken.

The protocol used for damage simulation with particles was adapted from previously described studies [15,16]. In brief, the PEEK or CoCr plate was clamped onto a sliding table mounted on the platen of an Instron materials testing machine (Instron, MA, USA). The PMMA cement particles described above were trapped (in excess) between a UHMWPE pin (3 mm diameter contact face) and the plate; before a load of 120 N was applied axially through the pin. Then, using the Instron materials testing machine, the plate was pulled beneath the pin for 15 mm at a speed of 8 mm/s to create third body damage. Five regions of damage were created on each plate with a spacing of 3 mm, the particles were passed over the plate 5 times in each region of damage. Third body damage was created perpendicular to the direction of the subsequent wear test (Figure 1a).

#### 2.2.2. Damage Simulation: Third Body Damage Created Using a Diamond Stylus

To create a scratch in the plates with a reproducible geometry, the plate was set up on a sliding table as described for the particle method, a stylus with a 200 µm radius diamond tip was axially loaded and the plate pulled beneath the stylus to create scratches. On each plate, 5 scratches were created running perpendicular to the direction of the wear study (Figure 1a). The lip height of the scratches was adjusted to either 1, 2 or 4 µm by changing the load applied to the stylus (Table A1 in Appendix A) [18].

#### 2.2.3. Pin-on-Plate Wear Simulation

Wear simulation was carried out using a six-station multi-axial pin-on-plate reciprocating rig and aimed to replicate the average contact pressure and cross-shear ratio in a total knee replacement [19]. The plate was mounted in a lubricant-containing bath, which reciprocated over a length of 20 mm at 1Hz, and as the bath reciprocated, the UHMWPE pin rotated (±20°) via a rack and pinion mechanism. A constant axial load of 160 N was applied through the pin (8 mm flat contact face) to give an average contact pressure of 3.18 MPa consistent with previous wear simulation of the UHMWPE-on-PEEK bearing couple [8]. All wear simulation studies were carried out using 25% bovine serum supplemented with 0.03% sodium azide solution as a lubricant and were carried out under rig running (room) temperature conditions as previously described and optimised for the UHMWPE-on-PEEK bearing couple [8]. Prior to the start of the study, the UHMWPE pins were soaked for a minimum of 2 weeks to maximise water uptake prior to cleaning and weighing using an XP26 digital microbalance (Mettler Toledo Inc., OH, USA) with a resolution of 1 µg. Measurements of each pin were taken until 5 consecutive measurements were within ± 5 µg and an average of these 5 measurements taken. Studies were carried out for 1 million cycles (MC) with gravimetric analysis of the UHMWPE pins every 0.3 MC. The weight loss of the pins was converted to a volume loss (V) using two unloaded soak controls to compensate for the uptake of moisture by the polyethylene and a density of 0.934 mg/mm^3^ for GUR 1020 UHMWPE. The wear factor (k) was calculated using the sliding distance for the test (X) and the applied load (P) as shown in Equation (1).

k = V/PX,(1)

For both methods of damage simulation, the wear of the UHMWPE pins against the plates which had undergone damage simulation was compared to that against undamaged, polished plates which served as a control. In the second approach (scratching using a diamond stylus), these plates are referred to as 0 µm lip height.

The surface topography of the plates was assessed pre- and post-test using a contacting Form Talysurf (Taylor Hobson, Leicester, UK) with a 2 µm conical tipped stylus. To analyse the surface roughness, a Gaussian filter was applied to the measurements and a 0.25 mm upper cut-off used as described in the ISO standard [20]. The surface roughness parameter of interest was the mean surface roughness (Ra). Measurements were taken both perpendicular to the direction of damage simulation (A) and perpendicular to the direction of the wear test (B) as shown in Figure 1a. The lip height (Figure 1b) of the scratches was also assessed by carrying out LS line form removal and primary analysis. For third body damaged plates, the density of the scratches within a given lip height range was calculated and expressed as the number of scratches per mm both after damage simulation and after the subsequent wear test. For this analysis, a threshold was applied whereby scratches with a lip height of <0.1 µm were not measured as they were considered too indistinct from the background topography of the plate to be reliably measured. For the plates scratched with a diamond stylus, the lip height of each scratch was measured at 5 points, scratches with a lip height less than 0.2 µm were not measured.

At the conclusion of the studies, images of the wear scars on the PEEK and CoCr plates were taken using an optical profiler, Alicona G5 IF (Graz, Austria) with 5× magnification.

A minimum of 3 sets of samples were used for each material configuration for each of the two approaches. The mean wear factor of the UHMWPE pins, the mean surface roughness (Ra) and the mean lip height of the scratches on the plates was determined with 95% confidence limits. Statistical analysis was carried out using a one-way ANOVA to compare the two configurations of UHMWPE-on-PEEK to UHMWPE-on-CoCr with significance taken at *p* < 0.05.

The data associated with this article is openly available from the University of Leeds Data Repository [18].

## 3. Results

### 3.1. Third Body Damage with PMMA Cement Particles

**Figure 2:**
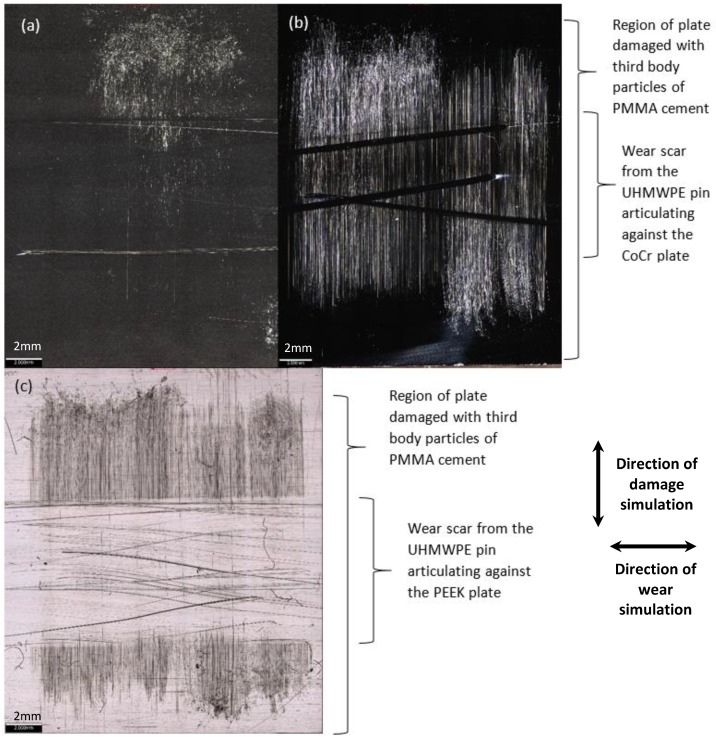
Representative images of the CoCr and PEEK plates following damage simulation with PMMA cement particles and wear simulation taken using an Alicona G5 IF with 5× magnification. (**a**,**b**) CoCr plates. In (**b**), scratches caused by the PMMA cement particles are more clearly visible; in both images, isolated scratches from the wear simulation can be seen. (**c**) A PEEK plate. The polishing and scratching from the wear simulation is visible running perpendicular to the direction of damage simulation. Scale bars represent 2 mm, arrows denote directions of damage simulation and wear simulation.

Having carried out third body damage simulation with PMMA cement particles, on the surface of all 3 PEEK plates, there was a high density of linear scratching visible in the region of damage; for CoCr, on one plate, scratching was visible, and on the other plates, there was little evidence of damage (Figure 2). Surface roughness measurements taken perpendicular to the direction of damage simulation (A) showed a significant increase of more than four-fold (*p* < 0.001) in mean surface roughness of the PEEK plates compared to CoCr (Table 1). Inspection of the plates showed no evidence of PMMA cement particles becoming embedded in the surface of the PEEK. 

In the control tests, after one MC wear simulation, the articulation of the pin against the polished plates caused discrete linear scratches on the CoCr plates and a higher density of scratches on the PEEK plates, resulting in a significantly higher post-test mean surface roughness of PEEK compared to CoCr (*p* = 0.03) (Table 1). Following wear simulation against the damaged plates, burnishing could be seen on the PEEK plates, where the scratches in the PEEK as a result of the damage simulation had been polished out leaving a defined wear scar (Figure 2c). However, within the wear scar, there was evidence of scratching from the articulation of the UHMWPE pin against the PEEK plate. On the CoCr plates, some discrete scratching was evident as a result of the wear simulation. At the conclusion of the study, there was no significant difference (*p* = 0.07) in the mean surface roughness of the PEEK and CoCr plates when measured either perpendicular to the damage simulation (A) or perpendicular to the direction of the wear test (B) (*p* = 0.09). However, despite there being no significant difference in the measurements, when measured perpendicular to the wear simulation, the mean surface roughness of the PEEK was much higher than that of CoCr (more than six-fold) with greater variability in the measurements. 

**Figure 3:**
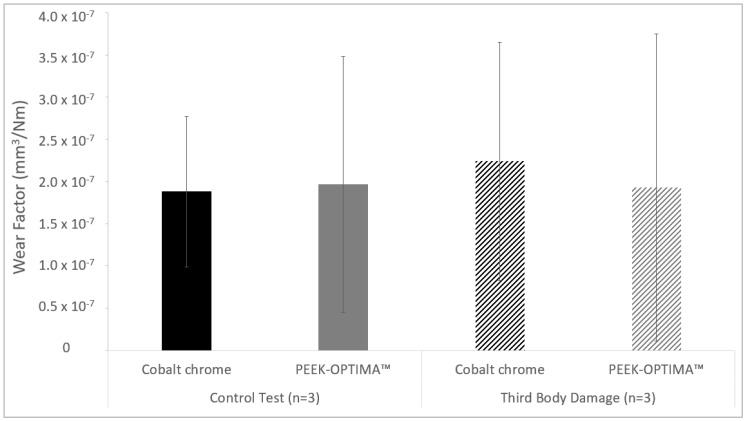
Mean wear factor (mm^3^/Nm) of UHMWPE pins (± 95% confidence limits) against smooth (control) CoCr and PEEK-OPTIMA™ plates and plates which had undergone damage simulation with particles of PMMA cement (n = 3).

In the control test, the mean wear factor with 95% confidence limits of the UHMWPE pins against smooth CoCr and PEEK plates was similar (*p* = 0.84) at 1.88 × 10^−7^ ± 0.92 × 10^−7^ mm^3^/Nm and 1.97 × 10^−7^ ± 1.52 × 10^−7^ mm^3^/Nm, respectively (Figure 3). Against third body damaged CoCr plates, the wear of the UHMWPE pins was 2.24 × 10^−7^ ± 1.41 × 10^−7^ mm^3^/Nm; against third body damaged PEEK plates, wear was 1.93 × 10^−7^ ± 1.82 × 10^−7^ mm^3^/Nm. There was no significant difference (*p* = 0.59) in the wear factors of the UHMWPE against the different material types and for all tests, the wear of the UHMWPE pins was linear. 

**Figure 4:**
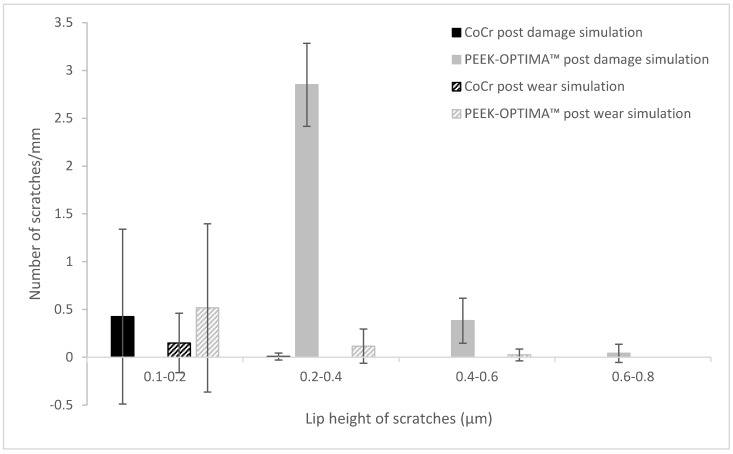
Mean number of scratches per mm (± 95 % confidence limits) within given lip height ranges on cobalt chrome and PEEK plates. Measurements taken post damage simulation and post wear test, (n = 3).

Analysis of the lip height of the scratches (Figure 4) showed that on the CoCr plates, lip heights were primarily in the 0.1–0.2 µm range both before and after damage simulation. For the PEEK plates, after damage simulation, most scratch lip heights were between 0.2 and 0.4 µm but some were as large as 0.8 µm, then after wear simulation, scratch lip heights were smaller and predominantly in the range of 0.1–0.2 µm.

### 3.2. Third Body Damage Created Using a Diamond Stylus

**Figure 5:**
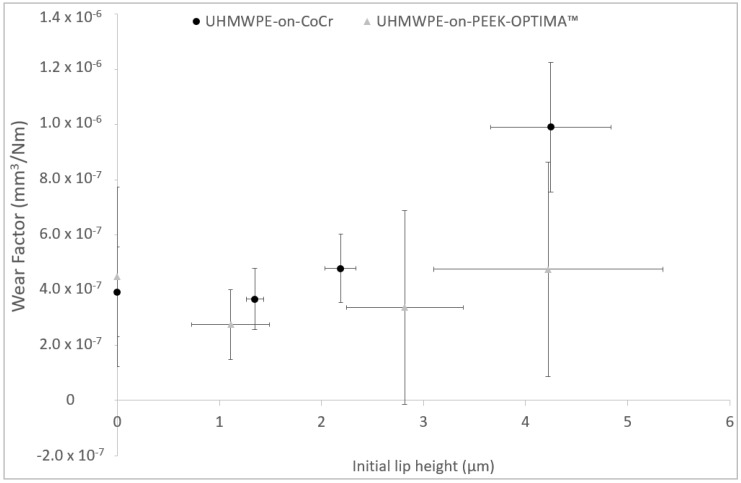
Mean wear factor (mm^3^/Nm) ± 95% confidence limits of UHMWPE pins tested against PEEK-OPTIMA™ and cobalt chrome plates scratched with a diamond stylus as a function of pre-test (initial) lip height, µm (± 95% confidence limits).

To adjust the lip height of the scratches created, the load applied to the diamond stylus was varied. To create scratches in the PEEK plates of the same magnitude as the CoCr, a lower load, approximately one-quarter of that required to create scratches in the CoCr was applied to the stylus (Table A1 in Appendix A) [18]. The magnitude of the lip heights created in the PEEK plates was more difficult to control than in the CoCr so variability in the lip heights was greater (Figure 5 and Table 2). 

**Figure 6:**
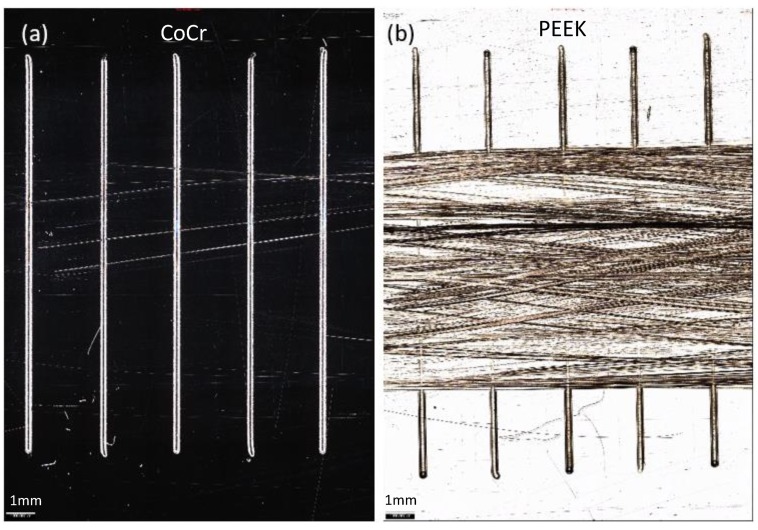
Representative images of CoCr (**a**) and PEEK (**b**) plates taken using an Alicona G5 IF with 5× magnification. The plates were scratched with a diamond stylus to create a scratch lip height of 4 µm, then 1 million cycles (MC) wear simulation was carried out perpendicular to the direction of the scratches. Scale bar represents 1 mm.

After 1 MC wear simulation, discrete scratches were visible in the wear scars on the CoCr plates; whereas a polished region was evident and there was a visible reduction in the lip height of the scratches in the PEEK plates (Figure 6). 

The mean wear factors of the UHMWPE pins against the scratched plates are shown in Figure 5. With no scratches on the plates (0 µm lip height), the wear of the UHMWPE against CoCr (3.4 × 10^−7^ ± 8.2 × 10^−7^ mm^3^/Nm) and PEEK (3.9 × 10^−7^ ± 5.3 × 10^−7^ mm^3^/Nm) was similar (*p* = 0.64); when scratches were created in the plates with a lip height of 4 µm, the wear of UHMWPE was significantly higher (*p* = 0.01) against CoCr than against PEEK at 9.7 × 10^−7^ ± 4.3 × 10^−7^ mm^3^/Nm and 3.8 × 10^−7^ ± 4.4 × 10^−7^ mm^3^/Nm, respectively, there were no significant differences (*p* > 0.05) in wear factor for the 1 and 2 µm lip height conditions. 

The lip heights of the scratches were assessed pre- and post-test. For the pre-test measurements, there was no significant difference (*p* < 0.05) between the lip height of the scratches in PEEK and CoCr for 1 or 4 µm. Post-test, the lip height of the scratches in the PEEK was significantly lower (*p* < 0.05) than in the CoCr for all scratch lip heights investigated due to the polishing effect of the pin against the plate (Table 2).

Surface roughness measurements taken perpendicular to the direction of wear simulation (B) between the scratches created with a diamond stylus are shown in Table 3. Pre-test, the mean surface roughness of the PEEK plates was significantly higher (*p* > 0.05) than the CoCr plates. After one MC wear simulation, there was a high density of linear scratching on the PEEK plates, and discrete scratches visible on the CoCr plates, resulting in a significantly higher (*p* < 0.05) mean surface roughness of the PEEK plates compared to CoCr.

In both studies, the PEEK plates were weighed at each measurement point; however, despite extensive pre-test soaking (>90 days in sterile water [21]), due to inconsistencies in moisture uptake by the PEEK [22], it was not possible to assess wear and the data was considered unreliable.

## 4. Discussion

The aim of this study was to investigate the influence of third body damage on the wear of UHMWPE-on-PEEK in a simple geometrical configuration. Two approaches were used to simulate third body damage: using particles of PMMA cement to create damage and scratching the implant surfaces directly with a diamond stylus prior to carrying out multi-directional pin-on-plate wear simulation against the damaged counterfaces. The wear of UHMWPE-on-PEEK was compared to UHMWPE-on-CoCr in studies carried out in parallel. It was hypothesised that the third body wear performance of UHMWPE-on-PEEK would differ to that of UHMWPE-on-CoCr.

Simulating third body damage with particles of PMMA cement caused light scratching on one of the three CoCr plates, which highlights the difficulty in controlling the particles trapped between the pin and plate to create reproducible third body damage using a particle method. Following damage simulation, the mean surface roughness of the plates was twice that reported by Cowie et al. [16] where third body damage simulation was carried out using a similar method. This higher surface roughness may be attributed to differences in the composition of the cement used. In the previous study, the cement used contained 10% barium sulphate as a radiopacifier, whilst in this study, the cement contained 14.75% zirconium dioxide. Whilst both barium sulphate and zirconium dioxide are commonly used additives to improve the visibility of bone cement under x-ray, previous third body wear simulation studies have shown the harder zirconium dioxide agglomerates to cause more damage to metallic counterfaces than barium sulphate [23]. This may account for the higher surface roughness of the plates following damage simulation in this study compared to previous work. Simulating third body wear with cement particles against PEEK plates gave a significantly higher mean surface roughness of the PEEK plates, which, therefore, indicates a reduced scratch resistance of PEEK compared to CoCr. Previous wear simulation studies of scratched metallic counterfaces have shown that it is the lip height of scratches in the metal that influences the wear of UHMWPE and that, to increase polyethylene wear, the scratch lip height must exceed a critical value [15,24]. When compared to control tests carried out against smooth plates, the damage simulation protocol used in this study did not generate scratches with a lip height of sufficient magnitude to influence the wear of UHMWPE against either damaged PEEK or CoCr plates. The wear factor of the UHMWPE pins in the control study was similar to that previously reported under comparable conditions [8]. It is interesting to note that the variability in the data was consistently larger in the UHMWPE-on-PEEK bearing couple compared to UHMWPE-on-CoCr and that following third body damage simulation, the variability in the data was further increased for both material combinations. This higher variability suggests that larger sample sizes and further testing under a wider range of conditions may need to be considered to fully understand the wear performance of the bearing couple.

Scratching the counterfaces with a diamond stylus allowed the geometry of the scratches to be more closely controlled. Previous studies have shown the lip height of scratches on retrieved hip and knee implants to be in the range of 0.01–4.1 µm [25,26] and experimental studies have shown larger scratch lip heights, up to 4 µm, to increase the wear of both hip [27] and knee implants [28]. To create a scratch of the same lip height in PEEK, a lower axial load was applied to the diamond stylus against the PEEK plates compared to CoCr due to the lower hardness of the PEEK polymer (Table A1) [18]. For both materials, there was a linear relationship between the load applied to the stylus and the lip height of the scratch created. Greater control of the lip height could be achieved in CoCr plates, where the scratch lip heights also had a more consistent geometry.

Against polished plates, there was no significant difference in the wear of UHMWPE against PEEK or CoCr. Scratches of 1 or 2 µm lip height in CoCr did not increase the wear of UHMWPE compared to polished control plates; however, with lip heights of 4 µm, the wear of UHMWPE was higher. This exponential relationship between scratch lip height and wear has previously been reported. Minakawa et al. showed scratches with lip heights below 0.5 µm to have a wear factor similar to that of polished plates; with larger lip heights (1 µm), a dramatic increase in wear factor was seen [15], and Lancaster et al. showed a similar relationship between Ra and wear factor [24]. In this study, a larger lip height (4 µm) was necessary to significantly increase the wear rate of the polyethylene, perhaps due to the differences in the grade of polyethylene used. When investigating the influence of scratch lip height in PEEK on the wear of UHMWPE, a different trend was seen. In the PEEK plates, a polishing effect of the pin against the plate was seen and the wear factor of the UHMWPE did not increase with lip height. When a 4 µm lip height was created in PEEK plates, the wear factor of the UHMWPE was significantly lower (*p* < 0.05) than when a scratch of similar magnitude was created in CoCr. This difference in wear performance can be attributed to the different hardness of the PEEK and CoCr materials.

Independent control tests were carried out for each of the damage simulation methodologies used and the different wear factors obtained may be attributable to the studies being carried out using different experimental rigs, slight differences in the composition of the cobalt chrome, the polymers used being from different batches and/or the studies being carried out by different researchers. It is, however, a strength of the study that independent control tests were carried out for each method.

There were a number of limitations that should be considered in the interpretation of this study. Firstly, this was a fundamental study carried out in a pin-on-plate configuration using simplified geometries of components and whilst the input kinematics replicated the average cross-shear and contact pressure in a total knee replacement [29]. The loading and motion profiles were simplified and consistent with previous studies [8], and it is not known whether the results would be replicated in a clinical setting. The components used were not sterilised or cross-linked; however, sterilisation by ethylene oxide, which is commonly used for TKRs, has been shown not to influence mechanical properties or induce crosslinking in polyethylene and, therefore, does not influence wear [30]. The sample size was small. In some cases, three repeats were carried out; however, it was deemed that a sufficiently large number of samples were tested to show the polishing effect of the UHMWPE pin against the PEEK plates scratched either with particles or a diamond stylus. This polishing effect did not occur in the UHMWPE-on-CoCr studies. It is also unclear whether the scratching on the PEEK plates after damage simulation or the polishing of the scratched regions of the plate by UHMWPE pins would contribute to an increase in PEEK wear or wear debris and whether in vivo this would accelerate osteolysis, as the inconsistent moisture uptake of PEEK made gravimetric analysis unreliable [8,22]. It is also likely that the relationship between scratch lip height and wear would be different for other grades or compositions of UHMWPE, particularly when articulating against CoCr or with the introduction of different kinematic conditions or geometries of components. Finally, this was a short-term study. When investigating damage simulation with particles, the particles were passed over the plate five times in each region of damage; and in the wear simulation, studies were limited to 1 MC.

## 5. Conclusions

Simulating third body damage with PMMA particles did not influence mean surface roughness enough to increase the wear of UHMWPE against either CoCr or PEEK. On the PEEK plates, a polishing effect of the UHMWPE pin against the scratched plate was observed.

When scratches with a lip height of greater magnitude were created directly on PEEK and CoCr plates, an exponential increase in UHMWPE wear factor was observed with increasing lip height against CoCr plates; whereas against PEEK plates, increasing lip height did not have a similar influence on the wear factor of UHMWPE.

## Figures and Tables

**Table 1:** Mean surface roughness (Ra), µm (± 95% confidence limits) of cobalt chrome and PEEK plates, pre-test, after damage simulation with polymethyl methacrylate (PMMA) cement particles and after wear simulation. Measurements were taken either perpendicular to or parallel to the direction of damage simulation (N = 3). Statistical analysis to compare ultra-high molecular weight polyethylene (UHMWPE)-on-PEEK to UHMWPE-on-CoCr, * denotes *p* < 0.05.

	Direction	Cobalt Chrome			PEEK-OPTIMA™		
		Pre-Test	After Damage Simulation with Particles	After Wear Simulation	Pre-Test	After Damage Simulation with Particles	After Wear Simulation
Control test	Perpendicular to damage simulation (A)	0.013 ± 0.001		0.014 ± 0.004	0.014 ± 0.001 *		0.019 ± 0.006
	Perpendicular to wear test (B)	0.013 ± 0.000		0.028 ± 0.030	0.019 ± 0.001 *		0.092 ± 0.159 *
Damage simulation	Perpendicular to damage simulation (A)	0.011 ± 0.014	0.012 ± 0.015	0.011 ± 0.009	0.006 ± 0.002	0.054 ± 0.006 *	0.018 ± 0.008
	Perpendicular to wear test (B)	0.011 ± 0.015	0.012 ± 0.016	0.014 ± 0.004	0.018 ± 0.001	0.023 ± 0.002 *	0.098 ± 0.164

**Table 2:** Mean lip height, µm (± 95% confidence limits) of scratches on PEEK-OPTIMA™ and cobalt chrome plates taken pre- and post- wear simulation (minimum N = 3). Statistical analysis to compare PEEK to CoCr, * denotes *p* < 0.05.

Lip Height (µm)	Cobalt Chrome		PEEK-OPTIMA™	
	Pre-Test	Post-Test	Pre-Test	Post-Test
1	1.35 ± 0.09	1.23 ± 0.16	1.11 ± 0.38	0.66 ± 0.66 *
2	2.19 ± 0.15	1.99 ± 0.16	2.82 ± 0.57 *	1.31 ± 0.70 *
4	4.25 ± 0.59	4.13 ± 0.44	4.22 ± 1.12	1.79 ± 1.02 *

**Table 3:** Mean surface roughness (Ra), µm (± 95% confidence limits) of PEEK-OPTIMA™ and cobalt chrome plates taken perpendicular to the direction of wear simulation (B) pre- and post- wear testing. Statistical analysis to compare PEEK to CoCr, * denotes *p* < 0.05.

Lip Height (µm)	Cobalt Chrome		PEEK-OPTIMA™	
	Pre-Test	Post-Test	Pre-Test	Post-Test
0	0.004 ± 0.001	0.027 ± 0.031	0.019 ± 0.004 *	0.540 ± 0.236 *
1	0.004 ± 0.001	0.008 ± 0.004	0.030 ± 0.012 *	0.729 ± 0.327 *
2	0.004 ± 0.001	0.019 ± 0.016	0.025 ± 0.007 *	0.616 ± 0.506 *
4	0.004 ± 0.001	0.019 ± 0.026	0.025 ± 0.008 *	0.839 ± 0.519 *

## Appendix A

**Table A1:** Load applied to a 200 µm radius diamond stylus to create scratches of a known geometry in PEEK and cobalt chrome plates.

Intended Lip Height of Scratches	PEEK-OPTIMA™		Cobalt Chrome	
	Weight Applied to Diamond Stylus (Kg)	Actual Lip Height of Scratches (µm)	Weight Applied to Diamond Stylus (Kg)	Actual Lip Height of Scratches (µm)
1 µm	0.51	1.11 ± 0.38	2.08	1.35 ± 0.09
2 µm	0.71	2.82 ± 0.57	2.99	2.19 ± 0.15
4 µm	1.02	4.22 ± 1.12	4.90	4.25 ± 0.59
